# Characterization of polymorphisms of genes ADH2, ADH3, ALDH2 and CYP2E1 and relationship to the alcoholism in a Colombian population

**Published:** 2015-12-30

**Authors:** Claudia Méndez, Mauricio Rey

**Affiliations:** Clinical Genetic Group, Facultad de Medicina, Instituto de Genética, Maestría en genética Humana, Universidad Nacional de Colombia, Bogotá, Colombia.

**Keywords:** Alcohol, alcoholism, alcohol dehydrogenase (ADH), aldehyde dehydrogenase (ALDH2), cytochrome P450 2E1 (CYP2E1)

## Abstract

**Objective:**

Identify and characterize polymorphisms of genes *ADH2*, *ADH3, ALDH2* and *CYP2E1* in a Colombian population residing in the city of Bogotá and determine its possible relationship to the alcoholism.

**Methods:**

*ADH2, ADH3, ALDH2,* and *CYP2E1* genotypes a population of 148 individuals with non-problematic alcohol and 65 individuals with alcoholism were determined with TaqMan probes and* P*CR-RFLP. DNA was obtained from peripheral blood white cells.

**Results:**

Significant difference was found in family history of alcoholism and use of other psychoactive substances to compare alcoholics with controls. When allelic frequencies for each category (gender) were considered, frequency of A2 allele carriers in *ADH2* was found higher in male patients than controls. In women, the relative frequency for c1 allele in *CYP2E1* was lower in controls than alcoholics. The *ALDH2* locus is monomorphic. No significant differences in allele distributions of the loci examined to compare two populations were observed, however when stratifying the same trend was found that these differences tended to be significant.

**Conclusions:**

This study allows us to conclude the positive association between family history of alcoholism and alcoholism suggesting that there is a favourable hereditary predisposition. Since substance dependence requires interaction of multiple genes, the combination of genotypes *ADH2* * *2, CYP2E1* * *1* combined with genotype homozygous *ALDH2* * *1* found in this study could be leading to the population to a potential risk to alcoholism.

## Abstract (es)

**Objetivo:**

Identificar y caracterizar los polimorfismos de los genes *ADH2, ADH3, ALDH2 *y *CYP2E1* de colombianos residentes en la ciudad de Bogotá y determinar su posible relación con el alcoholismo.

**Métodos:**

Se determinaron los genotipos *ADH2, ADH3, ALDH2* y *CYP2E1* a una población de 148 individuos con un consumo no problemático de alcohol y 65 individuos con alcoholismo. La genotipificación se realizó con sondas TaqMan y PCR-RFLP, el ADN se obtuvo de células blancas de sangre periférica.

**Resultados:**

Se encontró diferencia significativa en la historia familiar de alcoholismo y el uso de otras sustancias psicoactivas. Cuando se consideraron frecuencias alélicas para cada categoría (género), la frecuencia de portadores del alelo A2 en *ADH2* se encontró mayor en los pacientes masculinos que los controles. En las mujeres, la frecuencia relativa para el alelo C1 de *CYP2E1* fue menor en controles que en alcohólicos. El locus *ALDH2* es monomórfico. No se observaron diferencias significativas en las distribuciones alélicas de los loci examinadas al comparar las dos poblaciones, sin embargo al estratificar las mismas se encontró una tendencia a que esas diferencias fueran significativas.

**Conclusiones:**

Este estudio nos permite concluir la asociación positiva entre historia familiar de alcoholismo y el alcoholismo, lo que sugiere que existe una predisposición hereditaria favorable. Dado que la dependencia de sustancias requiere la interacción de múltiples genes como *ADH2* * *2*, *CYP2E1* * *1* combinado con el genotipo homocigótico *ALDH2* * *1* hallados en este estudio podría estar llevando a la población a un riesgo potencial hacia el alcoholismo.

## Introduction

Excessive alcohol consumption is one of the public health problems of greater magnitude relative to psychoactive legal use, being a problematic global threatening both individual development, family life and social life of a person. It is estimated that worldwide about 2,600 million people consume alcohol either occasionally, habitual, abusive or addictive; every year 2.5 million people die from alcohol-related causes 1.

Individuals who start early drug abuse constitute the group most at risk of developing an addiction. If predisposition could recognize at an early stage by means of a genetic profile could be directed toward prevention intervention in this population and even in later stages could propose alternative psychic or drug treatment according to genetic profile and/or patterns particular gene expression.

Alcohol is primarily metabolised in the liver through various metabolic pathways in which involving oxidative and no oxidative reactions. The main ethanol biotransformation reaction is catalysed by alcohol dehydrogenase (ADH) and is performed in the cytoplasm, where this enzyme oxidises the ethanol producing acetaldehyde, which in turn is oxidized by the enzyme aldehyde dehydrogenase (ALDH) to acetate. The second way of metabolism of ethanol is called ethanol inducible, a microsomal system called microsomal-ethanol oxidizing-system (MEOS), the major enzyme of this pathway is a cytochrome P4502E1 (CYP2E1), and it shows low affinity for ethanol compared with ADH and is responsible for a small amount (10%) of ethanol oxidation to acetaldehyde after alcohol intake moderate. It postulated that variations in genes of these enzymes may influence alcohol consumption, tissue damage related to the consumption and dependence 2.

The variation in alcohol elimination and the oxidation of acetaldehyde, genetically determined alcohol dehydrogenase 1-3 (before named ADH1A, ADH1B and ADH1C) and aldehyde dehydrogenase (ALDH2) plays a major role in the pathogenesis of the clinical syndrome. High concentrations of acetaldehyde in blood lead to unpleasant reactions such as flushing, tachycardia, nausea and others when drinking alcohol 3,4.

It has found three allelic variants of *ADH2 *locus *(ADH1B): ADHB* * *1* wild allele with G located in exon 3 that codes for an protein subunit containing in the position 48 is an arginine (CGC); *ADH1B* * *2* allele variant in which G changes to A replacing arginine in position 48 by a histidine (CAC) and finally *ADH1B* * *3* allele is a variant that codes for a protein subunit in which changes an arginine for cysteine at position 369. Above variants of *ADH1B (ADH2)* encode subunits ß1, ß2 and ß3 respectively. The product of allele *ADH1B* * *2* contains an atypical ß2 subunit with catalytic activity oxidation of ethanol 100 times greater than the product of *ADH1B* * *1* allele. *ADH1B* * *3* is found in populations African origin (20%), and rare in others. In both ß2 and ß3 subunits, substitution occurs in amino acid that makes contact with the coenzyme NAD^+^, giving that result a 70-80 times increase in the rate of enzyme yield compared with the ß1 subunit because the coenzyme is rapidly released at the end of the reaction. Both ß2ß2 and ß3ß3 show speeds of ethanol oxidation 30-40 times greater than ß1ß1 4.

In exon 8 of the gene *ADH1C* is located allele wild *ADH1C* * *1* (A) that codes for a protein subunit that position 350 has an isoleucine (ATT), the presence of a *ADH1C* * *2* polymorphism known in which there is a change an A/G, leads to a substitution in the protein subunit isoleucine 350 by a valine (GTT). The subunit &gamma;1 encoded by *ADH1C* * *1* was characterized by a maximum speed twice than &gamma;2 subunit encoded by *ADH1C* * *2*. The allele encoding protein ADH1C^*^1 is found in 90% of world population, while ADH1C^*^2 predominant in about 70% of the population of Far East. The allele ADH1C^*^1 predominates in Asian and African people by 90% while the allele frequency in Caucasians is 50% 5.

A substitution G/A in gene *ALDH2* generates atypical allele *ALDH2* ^*^ *2* that codifies a protein in which glutamic acid of position 504 is replaced by lysine. Allele *ALDH2* * *2* codifies for an enzyme with Km low for NAD^+^ and a Vmax reduced compared with the enzyme type wild giving as result a catalytic activity loss or null 6,7. The frequency of allele *ALDH2* * *2* it finds around a 40% in the populations of the far east of Asia and is less frequent in alcoholic than in the controls of different studies 8.

In the gene *CYP2E1* one of the polymorphisms more studied is transition C/T which presence alters the transcriptional activity of the gene because it´s located in the 5' region regulator of the transcription 9. Some studies have suggested the allele c2 (*CYP2E1* * *5B*) is associated to transcriptional activity of the gene up to 10 times superior to the common allele c1 (*CYP2E1* ^*^5A). This polymorphism is characterized by the loss of the site of cuts for the enzyme of restriction *RsaI* GTAC/GTAT when the allele behaves c2 10. The frequency of the allele c2 is markedly low in Caucasian and black populations of the United States of North America (1-5%) compared with the Asians (25%) 3. The genic variants homozygotes for *ADH2* * *2, ADH3* * *1* and *CYP2RB* digested by *RsaI c2* are isoforms with great activity enzymatic, whereas polymorphism *ALDH2* * *2* is one isoform with loss or null activity, this suggests one modulation of the metabolism of the ethanol and acetaldehyde that they influence in the pharmacokinetics of the alcohol consumption that it takes to a possible protection or predisposition to the alcoholism in some populations like for example those of the Far Asia, Mexican, Israelite Asquenazí, Sefardites and Russian immigrants 3. The objective of the present study was to identify and to characterize the polymorphisms of the genes of the alcohol dehydrogenase, aldehyde dehydrogenase and the cytochrome p450 2E1 in a population of residents in Bogotá and to determine its possible relation with the alcoholism.

## Material and Methods 

### Subjects 

The sample size was calculated using EpiInfo^®^ program (unpaired case-control), with α value of 0.05, and 95% confidence interval OR. To set the frequency the results of a study by Konishi *et al*., was taken as basis, study with Mexico-American men 3.

Initially the study was proposed as a comparative study between two populations, sporadic alcohol consumption (controls) and alcoholics (cases). The participants of the study were 291 subjects living in Bogota city. The 65 subjects belong to rehabilitation's foundations which previously had been diagnosed like poliaddicts by medical staff at each foundation, where the consumption problematic of alcohol was it first addiction which we call alcoholics. Were used the DSM-IV criteria for the definition of problem drinking. The other 226 participants were from colleges, universities and companies who helped us kindly. To all of them were applied questionnaire Alcohol Use Disorders Identification Test (AUDIT) like classification instrument 11. Of which 148 were classified like sporadic alcohol consumers (controls) and 78 of the possible controls had higher values at 8 in the AUDIT test and were grouped as problematic alcohol users (PAC, a third group).

The inclusion criteria were as follows: unrelated, first substance dependence was to alcohol, ability to give consent and signed consent forms. The exclusion criteria were as follows: hepatitis B and C, mood disorders and first substance dependence other than alcohol. The research protocol was approved by the ethics committee of the Faculty of Medicine, National University of Colombia.

Likewise all participants in the study answered a semi-structured interview of personal information as age, age of first consumption, family history, use of other psychoactive substances, type and quantity of beverage per day, health status and consumption habits. There were no dropouts in the study.

### Blood samples 

The DNA was obtained from cells of peripheral blood. Blood samples were collected in 5 mL tubes with EDTA anticoagulant. 300 μL were spotted on filter paper (Whatman 3MM CHR) for further processing. DNA from some of the samples was obtained according to the HotSHOT technique (Hot Sodium Hydroxide and Tris). In the Laboratory of Human Genetics of the Institute of Genetics of National University of Colombia the remaining sample was put under the process of DNA extraction for which use kit commercial (Kit Clean DNA Blood Isolation Kit Catalogue no. 12000-100 Mo-Bio). 

### Genotyping

The genotypes for the Alcohol dehydrogenase (*ADH2, ADH3*) and Aldehyde dehydrogenase (*ALDH2*) were determined through a collaborative study with The Indiana Alcohol Research Centre (ARC) of the Medicine Department of the University of Indiana using the TaqMan tests (Applied BioSystems, Foster City, CA). Each test contains two noticeable different TaqMan soundings with VIC or FAM like fluorescent markers which are united of preferential way to one or the other allele. Each reaction contains 5 μL of Universal Mastermix TaqMan 2X, 3.75 μL of water, 0.25 μL of Assay Mix 40X and 1 μL of sample. Controls were including in first positions of 96 wells plate: 2 negative controls, 2 or 3 samples of heterozygotes genotypes and 2 or 3 samples of each one of the homozygotes genotypes. Was used the termociclator MJ Research PTC-200. The products of PCR were analyzed in the System of Detection of Sequence (SDS-ABI PRISM^®^ 7300) 12.

The genotypification of gene *CYP2E1 *was carried out in Laboratory of Human Genetics of the Institute of Genetics of Universidad Nacional de Colombia. The amplification of gene *CYP2E1* work to a volume of 25 μL that contains 200 ng of genomic DNA, Buffer 1X, 200 μM of each dNTP, 2.5 mm MgCl_2_, 1.0 Unit of Taq polymerase and 10 picomol of each primer (foward: 5'-AACCAATGACTTGCTTATGT-3' and revers: 5'-CTTTCATGTATTAAGCATTTTCT-3'). Conditions of termocycling 1 cycle (95° C by 5 min.), 35 cycles (95° C by 5 s, 50.5° C by 30 s, 72° C by 1 min.), 1 cycle (72° C by 7 min, 4° C). The amplified ones were separated in 2% of agarose. Was used the Marker of weight (Gene Ruler 100 bp DNA Ladder, You ferment). These PCR products were digested with the RsaI enzyme (Fermentas and Invitrogen) under the following conditions: 6 μL of the mixture of PCR, 9.0 μL of molecular grade water, 1.0 μL of assay buffer 10X, 1.0 RsaI enzyme. The digestion was carried out during 3 h to 37° C. The enzyme was inactivated by incubation to 80° C by 20 min. The fragments were visualized in a gel of 3% of agarose. The presence of WT allele generate the site of cuts for this enzyme (GTAC), giving like result two bands, one of the 218 bp and other of 175 bp. When the allele loses the restriction site was observed a band of 393 bp. 

### Statistical analysis 

For the estimation of the allelic and genotypic frequencies of the genes of enzymes alcohol dehydrogenase, aldehyde dehydrogenase and the cytochrome p450 2E1 were used the program of genetic data analyses GENEPOP^®^ version 3.4 13. The relation of genotype and allelic frequencies was evaluated using a test of χ² and a value for *p* <0.05 was considered like statistically significant. The *p* value is from the chi-square or Fisher´s exact test.

## Results 

Age, years of first use of alcohol, family history and use of other psychoactive substances are presented in Table 1. The bigger percentage of the control population as the alcoholics were in ages within the 18-39 that is equivalent to a 78% of the total population. Significant difference (*p* <0.05) was found in family history of alcoholism and use of other psychoactive substances. Additionally individuals were discriminated by gender and AUDIT score.

**Table 1.** Distribution of individual characteristics of the sample, alcoholics and controls.

	Women				Men			
	Controls^*^	Alcoholics^*^	95% CI	*p*	Controls^*^	Alcoholics^*^	95% CI	p**
Age (yrs)	27.7±9.4	32.8±11.5	-9.905 to -0.295	0.0378	31±11.6	38±13.0	-11.522 to -2.478	0.0027
Age of first use(yrs)	19.6±10.2	18.3±6.1	-3.132 to 5.732	0.5612	18.3±8.9	16.5±6.6	-0.548 to 4.148	0.1317
	%	%			%	%		
Family history of alcoholism	38.0	96.0		<0.0001	26.4	97.0		<0.0001
Use of other sychoactive substances	11.5	83.0		<0.0001	15.0	75.6		<0.0001

^*^mean/standard deviation

^* *^ *p*-value from the independent t-test.

Participants completed a survey whose results are summarized below. As far as the familiar antecedents the 96% of women with alcoholism and 97% of the alcoholism´s men report to have some relative with problems of alcoholism (*p* <0.05), for both cases more related father and uncle. To the question about the consumption of other psychoactive substances 83% of women with alcoholism and 75% of men with alcoholism responded affirmatively (*p* <0.05). The desired substances more for the alcoholic group are the marijuana, cocaine, crack, cigarette, pills and poppers. The age of onset of alcohol consumption is similar in all groups being slightly younger the onset of alcoholic men and women. 

The allelic and genotypic frequencies of genes encoding *ADH2, ADH3, ALDH2* and *CYP2E1* in subjects with alcoholism, consumption problematic of alcohol (PAC) and control subjects are shown in Tables 2, 3, 4, and 5. In the group of alcoholics and in the group of problematic alcohol consumption compared to controls none of the observed differences reached statistical significance. When the study subjects were categorized according to their gender, the distribution of the genotypes and allele frequency did not differ significantly in both men and women (alcoholics vs controls) (Tables 4 and 5). However when allelic frequencies for each category (gender) were considered, frequency of A2 allele carriers in *ADH2* was found higher in male patients than controls (6.9% vs. 12.2%; *p*= 0.1571) (Table 5). In women, the relative frequency for c1 allele in *CYP2E1* were 89.3 and 97.9% for controls and alcoholics respectively (*p*= 0.067) (Table 4). None of the observed differences reached statistical significance. Individuals who's the group presented an AUDIT score between 20-40 (PAC) showed none significant differences in gene frequencies for all loci compared with the group with a non-problematic use of alcohol (controls). However when compared with the group of alcoholics can be seen close to the significant differences in the genes *ADH2* and *CYP2E1* (Table 3). 

**Table 2.** Allelic and genotypic frequencies of four alleles in subjects alcoholics (n= 65) and with no problematic alcohol consumption (Controls, n= 148).

Group	Genotypic^‡^				Gene^‡^	
	^*^1/^*^1	^*^1/^*^2	*p*	^*^2/^*^2	^*^1	^*^2	*p*
ADH2							
Controls	83.8 (124)	15.5 (23)		0.68 (1)	91.6 (271)	8.4 (25)	
Alcoholics	77.0 (50)	23.0 (15)	0.4943	0 (0)	88.5 (115)	11.5 (15)	0.3137
ADH3							
Controls	53.0 (78)	39.0 (58)		8.0 (12)	72.3 (214)	27.7 (82)	
Alcoholics	49.0 (32)	43.0 (28)	0.8676	8.0 (5)	70.8 (92)	29.2 (38)	0.7470
ALDH2							
Controls	100 (296)	0		0	100 (296)	0	
Alcoholics	10 (130)	0	^*^^*^	0	100 (130)	0	^*^^*^
CYP2E1							
Controls	83.1 (123)	16.9 (25)		0 (0)	91.5 (271)	8.5 (25)	
Alcoholics	87.7 (57)	12.3 (8)	0.5184	0 (0)	93.8 (122)	6.2 (8)	0.4151

^‡^%(n): percentage (number)

^* *^Without value

**Table 3.** Gene and genotypic frequencies of four alleles in the group with problematic alcohol consumption (PAC) classified with AUDIT score the 20-40 (n= 78) compared with no problematic alcohol consumption (Controls, n= 148) and alcoholics (n= 65).

Group	Genotypic^‡^				Gene^‡^		
	^*^1/^*^1	^*^1/^*^2	^*^2/^*^2	*p*	^*^1	^*^2	*p*
ADH2							
Controls	83.8 (124)	15.5 (23)	0.68 (1)		91.5 (271)	8.5 (25)	
PAC	88.5 (69)	10.3 (8)	1.2 (1)	0.6531	93.6 (146)	6.4 (10)	0.4522
Alcoholics	77.0 (50)	23.0 (15)	0 (0)	0.1906	88.5 (115)	11.5 (15)	0.1300
ADH3							
Controls	52.7 (78)	39.2 (58)	8.1 (12)		72.3 (214)	27.7 (82)	
PAC	52.5 (41)	41.0 (32)	6.4 (5)	0.8873	73.1 (114)	26.9 (42)	0.8602
Alcoholics	49.2 (32)	43.1 (28)	7.7 (5)	0.9066	70.7 (92)	29.3 (38)	0.6654
ALDH2							
Controls	100 (148)	0	0		100 (296)	0	
PAC	100 (78)	0	0	^*^^*^	100 (156)	0	^*^^*^
Alcoholics	100 (130)	0	0	^*^^*^	100 (130)	0	^*^^*^
CYP2E1							
Controls	83.0 (123)	17.0 (25)	0		92.0 (271)	8.0 (25)	
PAC	81.0 (63)	19.0 (15)	0	0.7987	90.0 (141)	10.0 (15)	0.6774
Alcoholics	87.7 (57)	12.3 (8)	0	0.3716	93.8 (122)	6.2 (8)	0.2837

^‡^%(n)= percentage(number)

PAC: problematic alcohol consumption

^* *^Without value

**Table 4.** Gene and genotypic frequencies of four alleles in the group of women with originating problematic alcohol consumption of Institutions (alcoholics, n= 24) and the group with no problematic alcohol consumption (Controls, n= 61).

Group	Genotypic‡				Gene‡		
	*1/*1	*1/*2	*2/*2	p	*1	*2	p
ADH2							
Alcoholic	79.2 (19)	20.8 (5)	0 (0)		89.6 (43)	10.4 (5)	
Controls	80.4 (49)	18.0 (11)	1.6 (1)	0.8869	89.3 (109)	10.7 (13)	0.9600
ADH3							
Alcoholic	54.2 (13)	41.6 (10)	4.2 (1)		75.0 (36)	25.0 (12)	
Controls	52.5 (32)	34.4 (21)	13.1 (8)	0.7089	69.7 (85)	30.3 (37)	0.4897
ALDH2							
Alcoholic	100 (48)	0	0		100 (96)	0	
Controls	100 (122)	0	0	^*^^*^	100 (244)	0	^*^^*^
CYP2E1							
Alcoholic	95.8 (23)	4.2 (1)	0		97.9 (47)	2.1 (1)	
Controls	78.7 (48)	21.3 (13)	0	0.1110	89.3 (109)	10.6 (13)	0.0670

^‡^%(n)= percentage(number)

^* *^Without value

**Table 5.** Gene and genotypic frequencies of four alleles in the group of men with alcoholism (n= 41) and the group with No alcohol consumption problems (Controls, n= 87).

Group	Genotypic‡				Gene‡		
	*1/*1	*1/*2	*2/*2	p	*1	*2	p
ADH2							
Alcoholic	75.6 (31)	24.4 (10)	0		87.8 (72)	12.2 (10)	
Controls	86.2 (75)	13.8 (12)	0	0.218	93.1 (162)	6.9 (12)	0.1581
ADH3							
Alcoholic	46.3 (19)	43.9 (18)	9.8(4)		68.3 (56)	31.7 (26)	
Controls	52.9 (46)	42.5 (37)	4.6(4)	0.4884	74.1 (129)	25.9 (45)	0.3297
ALDH2							
Alcoholic	100 (41)	0	0		100 (82)	0	
Controls	100 (87)	0	0	^*^^*^	100 (174)	0	^*^^*^
CYP2E1							
Alcoholic	82.9 (34)	17.1 (7)	0		91.5 (75)	8.5 (7)	
Controls	86.2 (75)	13.8 (12)	0	0.8231	93.1 (162)	6.9 (12)	0.8248

^‡^%(n)= percentage(number)

^* *^without value

The genotype A1A1 and allele frequencies A1-100% for the *ALDH2* gene show that the population is monomorphic for this marker. Our population has no protective allele, i.e. it is has the risk allele. The allele and genotype frequencies were in HWE for all SNP´s and groups, except in the latter gene.

## Discussion 

The studies of alcoholism are important to facilitate the implementation of strategies of public policies focused on prevention, early diagnosis, and treatment with more efficient therapies. In this study, we analyzed the association of some polymorphisms of *ADH2*, *ADH3, ALDH2 *and *CYP4502E1* genes with AD in Colombian population resident in Bogota city.

The genotypic and allelic frequencies found in alcoholics and controls are similar to that of ethnically and culturally close groups reported in other studies 14-20. Significant differences were not demonstrated in allelic nor genotypic frequencies between these two unstratified groups for none of the four alleles. However we find that men with A2 allele of *ADH2* and female subjects with c1 allele of *CYP2E1* had a higher frequency in alcoholics compared to the controls. However, this association was not significantly high. Although the sample size was large enough to demonstrate a significant association, it seems that after stratification of participants the size became relatively small which resulted in such inconclusive results.

As in other disorders of conduct, the dependency is inherited polygenetically and each gene only explains a small percentage of the variance. This suggests that to have a predisposition allele it does not imply an elevated risk; the majority of the carriers cannot express the upheaval 21,22. It has been reported that the presence of homozygote genotypes for alleles *ADH2* * *1* and *ADH2* * *2* in individuals which are also carry of genotype *ALDH2 1/1* exhibit a very little increase of acetaldehyde in blood after the ingestion of 0.3-0.5 g/Kg of alcohol, this sample that the acetaldehyde cannot be involved in protection against the alcoholism in individuals with polymorphisms *ADH2*. In Asian countries it has been reported that the risk of alcoholism with ADH2 ß2ß2 and ALDH2 2/2 is 100 times more under which in individuals with *ADH2* ß1ß1 and *ALDH2 1/1* 3.

The product of allele *ADH1B* * *2* contains a subunit ß2 atypical with a catalytic activity of oxidation of the ethanol 100 times greater than the product of allele *ADH1B* * *1* 4 these findings suggest individual them with consumption no problematic of alcohol without familiar antecedents they could tender oxidise the alcohol slowly whereas individuals with no problematic alcohol consumption with familiar antecedents would tend to oxidise the alcohol more quickly. It has been reported that in white subjects, high levels of acetaldehyde and reddening have been found in individuals with positive familiar history for alcoholism that stops those that presents negative familiar history stops alcoholism 23.

In the group of alcoholic´s men and women appeared differences near the significance for locus *ADH2* and *CYP2E1*, specifically in the frequencies of allele c2 (*CYP2E1* * *5B*) which is associated to a transcriptional activity of the gene up to 10 times superior to the common allele c1 (*CYP2E1* * *5A*) that as well it relates at the risk of dependency 24,25. The increase of acetaldehyde could stimulate the presence of adverse effects in the individual inducing to stop the alcohol consumption.

In the present study, the combination of genotypes *ADH2, CYP2E1* and *ALDH2* could be taking to population to a possible risk, since presenting alleles whose presence allows a rate of greater oxidation (*ADH2* * *2, CYP2E1* * *1*) combined with the genotype homozygote *ALDH2* * *1* would increase the rates of explanation of the alcohol and acetaldehyde giving as result a greater tolerance to the consumption of great amounts of alcohol being this a risk factor to develop a dependency. Added previous the survey it showed that those people who also present a problematic alcohol consumption show a greater tendency to consume another type of psychoactive substances (*p *≤0.0001). The alcohol dependence frequently coexists with other addictions, in which includes the substance abuse, dependence nicotine, combined with psychiatric illness among those include depression, anxiety, antisocial personality, behaviour disorders among others. Similarly the positive association between family history of alcoholism and alcoholism (*p *≤0.0001) is clear, suggesting that there is a favourable hereditary predisposition.

The different gene association studies and alcoholism in the world have yielded conflicting results, our study was no exception. In addition, Colombian population was originated from the admixture of Spaniard colonists, African slaves and aboriginal populations, mainly during the XVI-XVIII Centuries. This admixture had different proportions in space and time, which made current Colombian population very heterogeneous, even among the accepted five geographic sub-regions and the city of Bogotá has been the epicenter of the meeting of these regions 26. Probably these conflicting results are occurring because failures in the study design, the variability in the selection of alcoholics, the nature of the controls, indicating the need for further studies to be replicated and correlated, especially in different ethnic groups. 

It is noteworthy that is recommended to expand the number of individuals, besides linking a larger number of participants different regions of Colombia in order to corroborate findings from this study and to estimate potential deference by place of origin and combine the study of biochemical tests that establish physiological reactions to corroborate the genetic findings. These markers of ethanol metabolism may be useful in addition to other markers of other routes such as those related to neuronal level rewards. For this reason it was decided to investigate other genes that could be related to alcohol addiction in Colombia, as are the mechanisms involved common neurobiological psychiatric illnesses and use other psychoactive substances, in which systems are serotonergic, gabaergic and dopaminergic among others 27

## Conclusions 

This study allows us to conclude the positive association between family history of alcoholism and alcoholism (*p *≤0.0001) is clear, suggesting that there is a favourable hereditary predisposition. Since substance dependence requires interaction of multiple genes, the combination of genotypes *ADH2* * *2, CYP2E1* * *1* combined with genotype homozygous *ALDH2* * *1* found in this study could be leading to the population to a potential risk, given that the alleles present in our population whose presence allows a higher oxidation rate, increase clearance rates alcohol and acetaldehyde, resulting in greater tolerance to the consumption of large quantities of alcohol being this is a risk factor for developing a dependency to alcohol.
